# Metabolic remodeling by RNA polymerase gene mutations is associated with reduced β-lactam susceptibility in oxacillin-susceptible MRSA

**DOI:** 10.1128/mbio.00339-24

**Published:** 2024-05-02

**Authors:** Shinya Watanabe, Chijioke A. Nsofor, Kanate Thitiananpakorn, Xin-Ee Tan, Yoshifumi Aiba, Remi Takenouchi, Kotaro Kiga, Teppei Sasahara, Kazuhiko Miyanaga, Srivani Veeranarayanan, Yuzuki Shimamori, Adeline Yeo Syin Lian, Thuy Minh Nguyen, Huong Minh Nguyen, Ola Alessa, Geoffrey Peterkins Kumwenda, Sarangi Jayathilake, Jastin Edrian Cocuangco Revilleza, Priyanka Baranwal, Yutaro Nishikawa, Feng-Yu Li, Tomofumi Kawaguchi, Sowmiya Sankaranarayanan, Mahmoud Arbaah, Yuancheng Zhang, Maniruzzaman ‌‌, Yi Liu, Hossain Sarah, Junjie Li, Takashi Sugano, Thi My Duyen Ho, Anujin Batbold, Tergel Nayanjin, Longzhu Cui

**Affiliations:** 1 Division of Bacteriology, Department of Infection and Immunity, Jichi Medical University, Tochigi, Japan; 2 Department of Biotechnology, School of Biological Sciences, Federal University of Technology Owerri Nigeria, Owerri, Nigeria; 3 School of Medicine, Jichi Medical University, Tochigi, Japan; 4 Research Center for Drug and Vaccine Development, National Institute of Infectious Diseases, Tokyo, Japan; Harvard Medical School, Boston, Massachusetts, USA; Western Sydney University, Penrith, New South Wales, Australia

**Keywords:** *Staphylococcus aureus*, MRSA, antimicrobial resistant, beta-lactams, OS-MRSA, oxacillin, RNA polymerases, rpoBC

## Abstract

The emergence of oxacillin-susceptible methicillin-resistant *Staphylococcus aureus* (OS-MRSA) has imposed further challenges to the clinical management of MRSA infections. When exposed to β-lactam antibiotics, these strains can easily acquire reduced β-lactam susceptibility through chromosomal mutations, including those in RNA polymerase (RNAP) genes such as *rpoBC*, which may then lead to treatment failure. Despite the increasing prevalence of such strains and the apparent challenges they pose for diagnosis and treatment, there is limited information available on the actual mechanisms underlying such chromosomal mutation-related transitions to reduced β-lactam susceptibility, as it does not directly associate with the expression of *mecA*. This study investigated the cellular physiology and metabolism of six missense mutants with reduced oxacillin susceptibility, each carrying respective mutations on RpoB^H929P^, RpoB^Q645H^, RpoC^G950R^, RpoC^G498D^, RpiA^A64E^, and FruB^A211E^, using capillary electrophoresis-mass spectrometry-based metabolomics analysis. Our results showed that *rpoBC* mutations caused RNAP transcription dysfunction, leading to an intracellular accumulation of ribonucleotides. These mutations also led to the accumulation of UDP-Glc/Gal and UDP-GlcNAc, which are precursors of UTP-associated peptidoglycan and wall teichoic acid. Excessive amounts of building blocks then contributed to the cell wall thickening of mutant strains, as observed in transmission electron microscopy, and ultimately resulted in decreased susceptibility to β-lactam in OS-MRSA.

**IMPORTANCE:**

The emergence of oxacillin-susceptible methicillin-resistant *Staphylococcus aureus* (OS-MRSA) strains has created new challenges for treating MRSA infections. These strains can become resistant to β-lactam antibiotics through chromosomal mutations, including those in the RNA polymerase (RNAP) genes such as *rpoBC*, leading to treatment failure. This study investigated the mechanisms underlying reduced β-lactam susceptibility in four *rpoBC* mutants of OS-MRSA. The results showed that *rpoBC* mutations caused RNAP transcription dysfunction, leading to an intracellular accumulation of ribonucleotides and precursors of peptidoglycan as well as wall teichoic acid. This, in turn, caused thickening of the cell wall and ultimately resulted in decreased susceptibility to β-lactam in OS-MRSA. These findings provide insights into the mechanisms of antibiotic resistance in OS-MRSA and highlight the importance of continued research in developing effective treatments to combat antibiotic resistance.

## INTRODUCTION

Methicillin-resistant *Staphylococcus aureus* (MRSA) is one of the most distributed antibiotic-resistant bacterial pathogens in clinical settings, posing a significant threat to human health around the world (1). The breakpoints for diagnosing MRSA in clinical microbiology laboratories are defined as an oxacillin minimum inhibitory concentration (MIC) ≥ 4 µg/mL (Clinical and Laboratory Standards Institute) or cefoxitin MIC ≥ 8 µg/mL (European Committee on Antimicrobial Susceptibility Testing). β-Lactam resistance in MRSA is commonly associated with the expression of penicillin-binding protein 2a (PBP2a; also known as PBP2′), a peptidoglycan transpeptidase with low affinity for β-lactam antibiotics (2–4). Although β-lactam resistance is primarily mediated by the *mecA* gene encoding PBP2a, clinical MRSA isolates usually exhibit varying levels of β-lactam resistance, the diversity of which cannot be simply explained by the expression of PBP2a and the presence of functional *mecA* and *bla* regulators (MecI/MecR1/MecR2 and BlaI/BlaR1) (5–8). Oxacillin-susceptible MRSA (OS-MRSA) and borderline oxacillin-resistant *S. aureus* strains, both carrying *mecA* gene but exhibiting susceptible or borderline resistance to oxacillin, have been isolated in clinical settings (8–15). Owing to their susceptibility to oxacillin, OS-MRSA might be misidentified as methicillin-susceptible *S. aureus* (MSSA) by routine diagnostic tests in most clinical microbiology laboratories where *mecA* and PBP2a detections are unavailable. These oxacillin-susceptible MRSA strains readily express high-level resistance (homogeneous resistance phenotype) following exposure to β-lactams due to the presence of the *mecA* gene, similar to clinical isolates with a low-level β-lactam resistance phenotype, which are frequently reported to show heterogeneous expression of methicillin resistance due to the presence of a small proportion of resistant bacteria among the populations sensitive to β-lactams (16–19) and consequently cause treatment failure with β-lactam antibiotics that are commonly used to treat MSSA infections (20).

The transition from low- to high-level resistance is complex because the shift is often caused by gene mutations or genetic rearrangements that are not directly relevant to the function of *mecA* (8, 18, 21–29). Among the genes responsible for β-lactam resistance, RNA polymerase (RNAP) genes, *rpoBC,* are frequently found mutated in laboratory-derived β-lactam-resistant mutants (8, 21, 25). RNAP is a DNA-dependent RNA polymerase that catalyzes RNA synthesis in all cellular organisms (30). In Gram-positive bacteria, a RNAP holoenzyme consists of four essential (α2ββ′) and four accessory (δεσω) subunits (31). Since RNAP is the major central transcriptional machinery in bacterial cells, mutations in any of the RNAP subunits can affect cellular activities and antimicrobial susceptibility. Mutations in the *rpoA* gene, which encodes the α subunit of RNAP, have been reported to alter the promoter recognition domain and lead to a change in cellular phenotype (32). Mutations in the *rpoB* and *rpoC* genes decrease RNAP fidelity (33) and, as the binding site of rifampicin is located in the β subunit of RNAP at the DNA:RNA binding cleft, the mutations of *rpoB* gene paralleled a block in RNA extension and caused rifampicin resistance (34). In *Mycobacterium tuberculosis*, *rpoB* mutations caused rifampicin resistance while increasing cell wall permeability and susceptibility toward vancomycin (35). Other than conferring β-lactam and rifampicin resistance, *rpoBC* mutations contributed to vancomycin resistance, autolysis activity, and slow growth in MRSA (36–39). Importantly, MRSA strains carrying *rpoB* mutations have been isolated from patients who underwent long-term antibacterial treatment, and the isolates were shown to be expressing increased β-lactam resistance (40, 41). Despite the extensive reports on the association between RNAP mutations and antimicrobial susceptibility, the molecular mechanisms of β-lactam resistance mediated by *rpoBC* mutations have not been fully understood.

Recently, we collected 43 genetically diverse clinical OS-MRSA strains, which could be classified into 11 MLST types and 4 different SCC*mec* types (8). Mutations associated with oxacillin susceptibility were identified by analyzing the whole genome of 100 mutants with reduced oxacillin susceptibility derived from 26 representative OS-MRSA strains of seven main phylogenetic clades (8). Among these mutants, *rpoBC* genes were found to be most frequently mutated regardless of the varied genetic backgrounds in the parent strains. Interestingly, we also found that the expression of *mecA* and PBP2a was not significantly correlated with the oxacillin MICs of these OS-MRSA-derived mutants, although the *mecA* gene is essential for high-level β-lactam resistance because *mecA* deletion in RpoC^P358L^, RpoB^G645H^, and RpoC^G498D^ mutants showed a decrease in oxacillin MIC from 4, 32, and 256 µg/mL, respectively, to 0.38 µg/mL, a level similar to that of *mecA*-knockout mutant JMUB217(∆*mecA*) (8). These findings emphasized the need for further investigation into the mechanisms underlying reduced oxacillin susceptibility in these mutants. In our present study, we aimed to unravel the mechanism associated with *rpoBC*-mediated acquisition of β-lactam resistance by investigating the cellular metabolisms of OS-MRSA-derived mutants with reduced oxacillin susceptibility. We focused on ribonucleotide metabolisms due to a clear downregulation of purine/pyrimidine biosynthesis and nucleotide transporter genes (*xprT*, *purF*, *guaAB*, *pyrRP*, and *hisIG*) observed in the transcriptome analysis of the representative *rpoBC* mutants with reduced oxacillin susceptibility (8).

## RESULTS

### Antibiotic susceptibility of mutants with reduced oxacillin susceptibility

**Fig 1:**
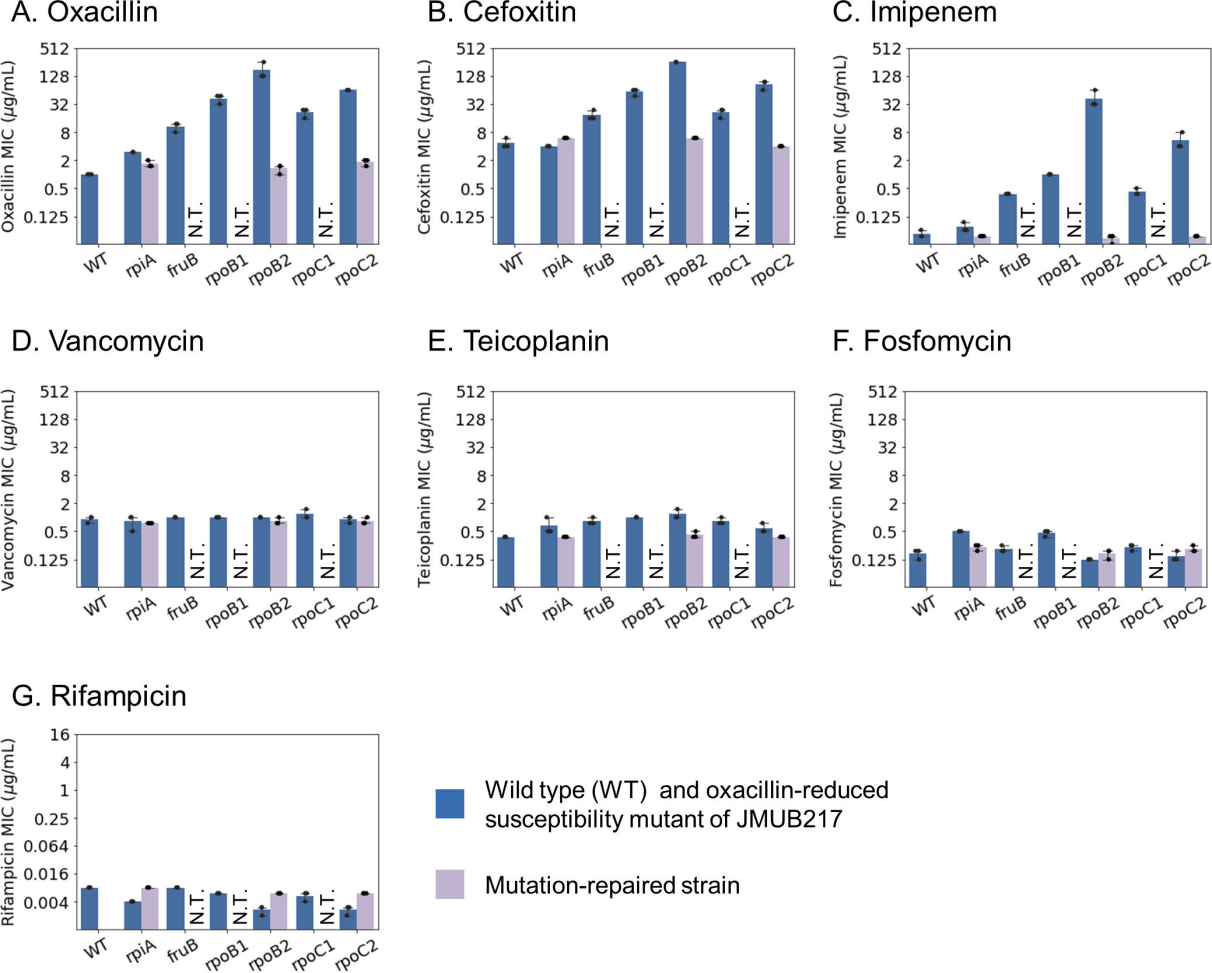
MIC of the JMUB217-derived mutants. MICs of eight drugs for six JMUB217-derived mutants with reduced oxacillin susceptibility: *rpiA* (JMUB217-7 RpiA^A64E^), *fruB* (JMUB217-13 FruB^A211E^), *rpoB1* (JMUB217-23 RpoB^H929P^), *rpoB2* (JMUB217-22 RpoB^Q645H^), *rpoC1* (JMUB217-19 RpoC^G950R^), and *rpoC2* (JMUB217-24 RpoC^G498D^) and mutation-repaired strains of JMUB217-7 (*rpiA*), JMUB217-22 (*rpoB2*), and JMUB217-24 (*rpoC2*) were determined. MICs were measured using E-test, and the graph represents the mean ± SD of biological triplicates.

Chromosomal mutations responsible for reduced oxacillin susceptibility in mutant strains derived from OS-MRSA were previously identified (8). These mutations were found to be distributed among 46 genes classified into various functional categories. To gain more insight into the molecular mechanisms regulating the decrease of β-lactam susceptibility in OS-MRSA, six representative mutants with reduced oxacillin susceptibility derived from the JMUB217 strain were further analyzed (8). JMUB217-7 (RpiA^A64E^), JMUB217-13 (FruB^A211E^), JMUB217-23 (RpoB^H929P^), JMUB217-22 (RpoB^Q645H^), JMUB217-19 (RpoC^G950R^), and JMUB217-24 (RpoC^G498D^) are included in this study because (i) single mutation was detected on their chromosomes, and (ii) RNAP genes (*rpoBC*), purine biosynthesis gene (e.g., *rpiA*), and glycolysis gene (e.g., *fruB*) were the most frequently mutated genes identified among mutants with reduced oxacillin susceptibility in our previous study (8). In addition, three mutation-repaired strains of JMUB217-7 (RpiA^A64E^), JMUB217-22 (RpoB^Q645H^), and JMUB217-24 (RpoC^G498D^) were constructed. MICs of eight antibiotic agents for all the JMUB217-derived mutants and mutation-repaired strains were determined. As described previously, the oxacillin MIC increased from 1.0 ± 0.0 µg/mL in wild-type strain to 3.0 ± 0.0, 11 ± 2.3, 43 ± 9.2, 171 ± 74, 21 ± 4.6, and 64 ± 0.0 µg/mL in JMUB217-7, JMUB217-13, JMUB217-23, JMUB217-22, JMUB217-19, and JMUB217-24, respectively (Fig. 1). Similar to oxacillin, the JMUB217-derived mutants exhibited increment in MIC of other β-lactam antibiotics, including cefoxitin and imipenem (Fig. 1), while their susceptibility against other cell wall synthesis inhibitors, such as vancomycin, teicoplanin, and fosfomycin, did not differ from wild-type JMUB217. On the other hand, the three mutation-repaired strains showed similar levels of MICs to the wild-type strains. We have also determined the rifampicin MIC of the mutants because it has been reported that mutations in *rpoB* increased rifampicin MIC (42, 43). However, all mutants with reduced oxacillin susceptibility were susceptible to rifampicin (Fig. 1).

### *mecA* and purine biosynthesis gene expressions of mutants with reduced oxacillin susceptibility

**Fig 2:**
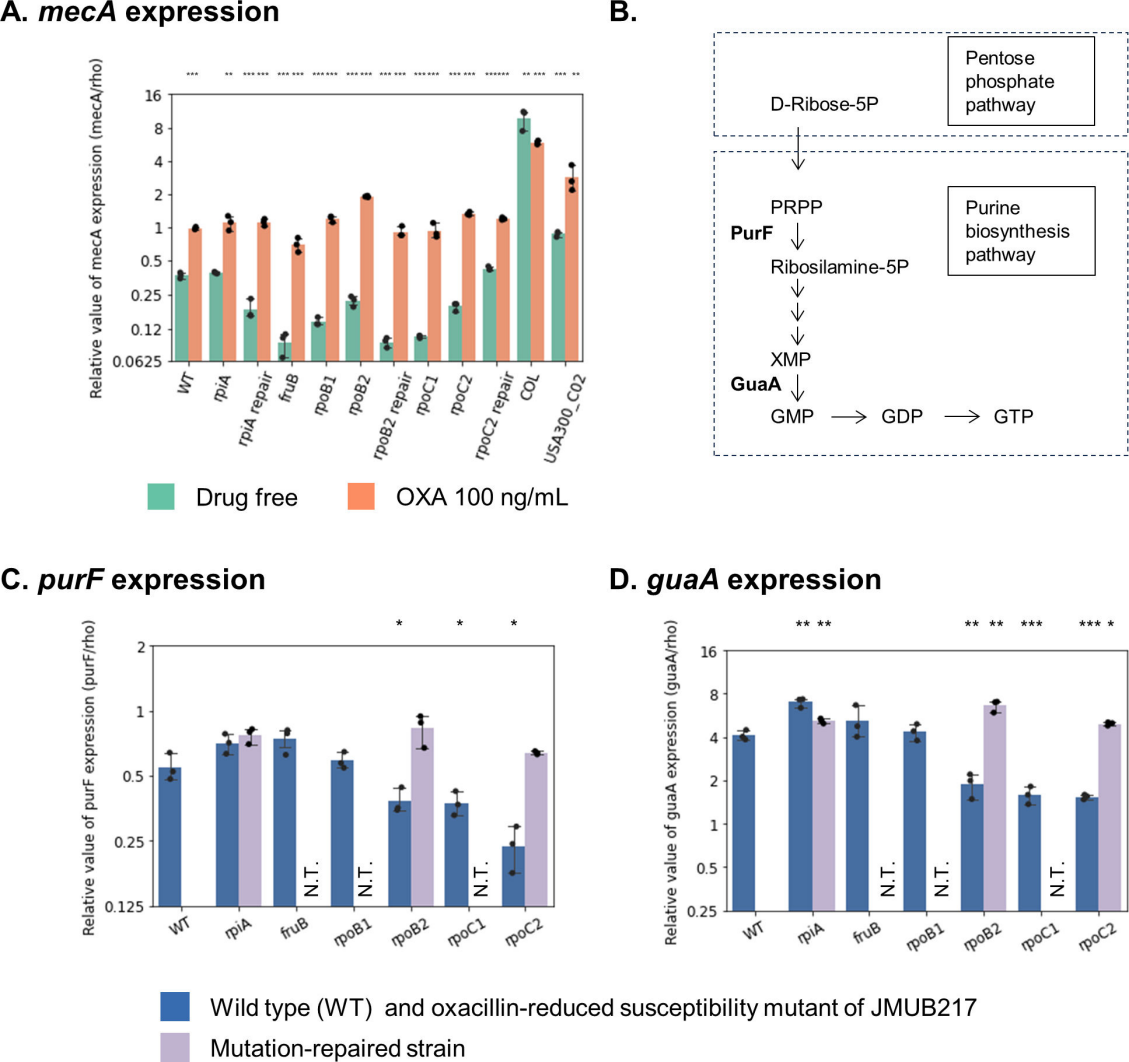
RNA expression levels of JMUB217-derived mutants. qRT-PCR results for (**A**) *mecA*, (**C**) *purF*, and (**D**) *guaA* in six JMUB217-derived mutants with reduced oxacillin susceptibility: *rpiA* (JMUB217-7 RpiA^A64E^), *fruB* (JMUB217-13 FruB^A211E^), *rpoB1* (JMUB217-23 RpoB^H929P^), *rpoB2* (JMUB217-22 RpoB^Q645H^), *rpoC1* (JMUB217-19 RpoC^G950R^), and *rpoC2* (JMUB217-24 RpoC^G498D^); three mutation-repaired strains (*rpiA*, *rpoB2,* and *rpoC2*); and two reference MRSA strains COL and USA300_C02 with or without exposure to 100 ng/mL oxacillin. The data are shown as means  ±  SD of three biological replicates. *, **, ***, and ns indicate *P* < 0.05, 0.01, 0.001, and not significant, respectively, given by the Student’s *t*-test. (**B**) Purine biosynthesis pathway of *S. aureus* JMUB217.

Expression of *mecA* and key enzymes of the purine biosynthesis pathway, *purF* and *guaA,* was determined by qRT-PCR (Fig. 2) to confirm our previous findings (8). *mecA* expression profiles of the six representative mutants with reduced oxacillin susceptibility and the three mutation-repaired strains were studied, with MRSA strains COL (ST256, SCC*mec* type I, oxacillin MIC > 256 µg/mL) and USA300_C02 (ST8, SCC*mec* type IVa, oxacillin MIC of 48 µg/mL) included in the analysis as MRSA reference strains. Our results showed that *mecA* expressions were not significantly increased in the six mutant strains, with or without oxacillin induction, compared with COL and USA300_C02 (Fig. 2A), indicating that their basal and induced *mecA* expression levels were lower than those of MRSA. On the other hand, both *purF* and *guaA* expressions, which were significantly downregulated in JMUB217-22 (RpoB^Q645H^), JMUB217-19 (RpoC^G950R^), and JMUB217-24 (RpoC^G498D^) (Fig. 2C and D), were rescued in the mutation-repaired strains of JMUB217-22 and JMUB217-24. These results indicated that the *rpoBC* mutations are mediating downregulations of the purine biosynthesis pathway, comparable to RNA-seq analysis in our previous study.

### Metabolic remodeling through oxacillin-induced mutations

**Fig 3:**
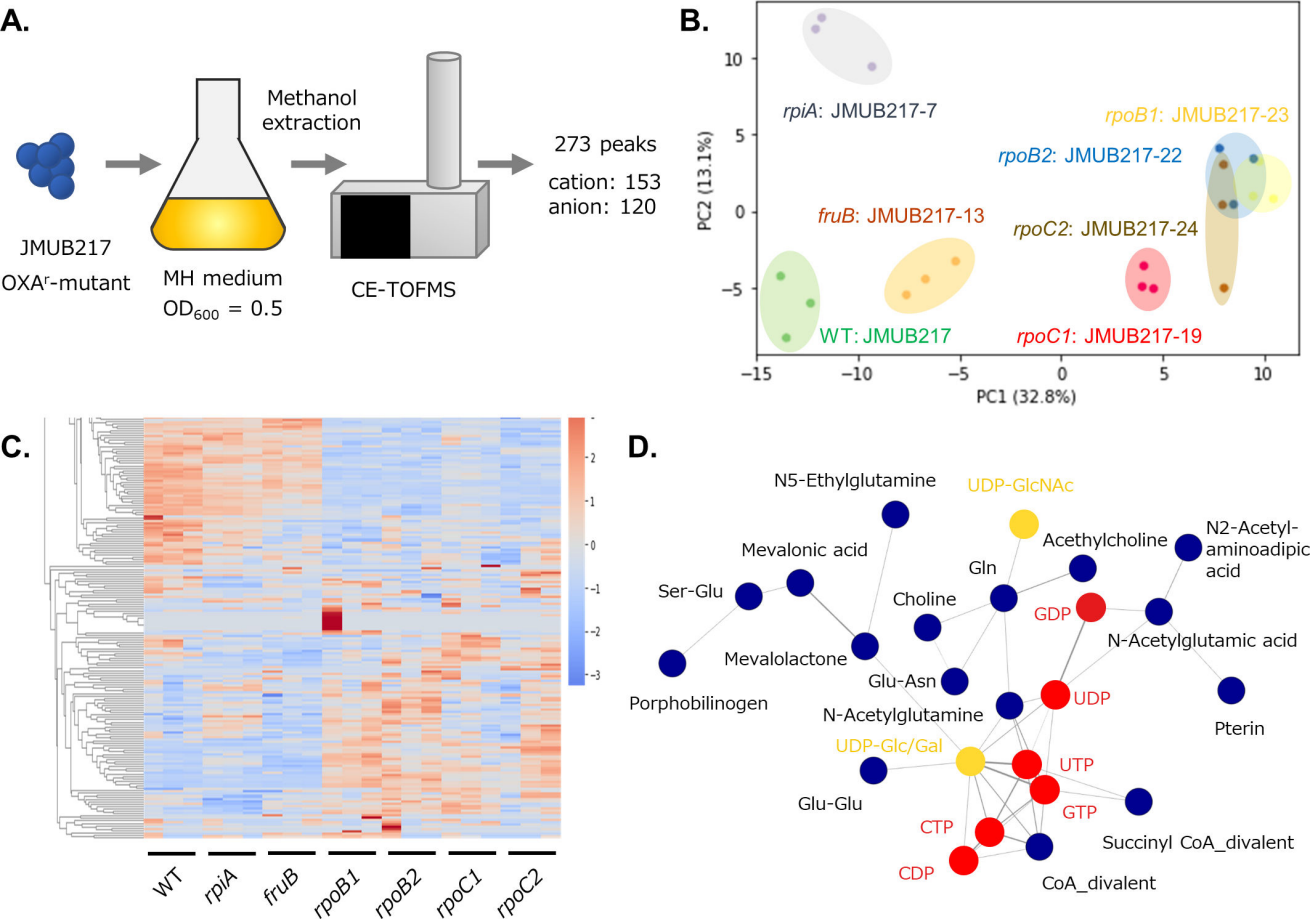
Metabolomic analysis of JMUB217-derived mutants. (**A**) Schematic diagram depicting the workflow of metabolomics analysis by CE-TOF-MS. (**B**) PCA score plot of metabolites in JMUB217-derived mutants with reduced oxacillin susceptibility: *rpiA* (JMUB217-7 RpiA^A64E^), *fruB* (JMUB217-13 FruB^A211E^), *rpoB1* (JMUB217-23 RpoB^H929P^), *rpoB2* (JMUB217-22 RpoB^Q645H^), *rpoC1* (JMUB217-19 RpoC^G950R^), and *rpoC2* (JMUB217-24 RpoC^G498D^). (**C**) Heat map representation of the metabolome analyzed by hierarchical clustering analysis. The distances between peaks are displayed in tree diagrams. Red and blue colors indicate higher and lower levels of metabolites, respectively. (**D**) Network analysis of the metabolites of JMUB217-derived mutants. Generally, metabolites are presented in blue circles, while ribonucleotides and their derivatives are highlighted in red and yellow, respectively. Figures 3 to 5 and Fig. S1 were constructed from data of the same metabolomics analysis.

Untargeted metabolomics was applied to study metabolic changes in the OS-MRSA strain JMUB217 and its six derivative mutants with reduced oxacillin susceptibility. Three biological replicates were prepared from the mid-log phase culture, and all samples were analyzed using the capillary electrophoresis time-of-flight mass spectrometry (CE-TOF-MS) for metabolomic profiling (44, 45). A total of 273 metabolite peaks (153 in cation and 120 in anion mode) were detected and annotated by the peak library (Fig. 3A). Detailed metabolomic profiles are presented in Table S1. Principal component analysis (PCA) of the metabolic data revealed clear clustering between mutated genes (Fig. 3B), with the first and second principal components (PC1 and PC2) accounting for 32.8% and 13.1% of the total variance, respectively. RNAP mutations were clearly correlated with PC1 because the metabolites of *rpoB* and *rpoC* mutated strains were positively aligned with PC1. Moreover, the PCA plots of the *rpoB* and *rpoC* groups partially overlapped with each other, indicating that the four different mutations in *rpoBC* affected the metabolism of *S. aureus* in a similar manner. This observation was supported by hierarchical clustering analysis of the metabolomics data, which showed a marked difference in the metabolic profiles between *rpoBC* mutants and wild-type or the other strains carrying *rpiA* and *fruB* mutations (Fig. 3C). The heat map has indeed identified a group of metabolites specifically accumulated in *rpiA*-mutated cells distinct from *rpoBC*- and *fruB*-mutated cells (Fig. 3C). To identify co-accumulated metabolites in *rpoBC*-mutated cells, network analysis was performed. The analysis revealed 15 clusters of co-accumulated metabolites in JMUB217 and its mutants with reduced oxacillin susceptibility. One of the clusters contains 24 metabolites, which were specifically accumulated in *rpoBC* mutants (Fig. 3D). The cluster includes (i) ribonucleoside-di/triphosphate (GDP, UDP, CDP, GTP, UTP, and CTP) and its derivatives (UDP-GlcNac, UDP-Glc/Gal); (ii) glutamine/glutamate and its derivatives (Gln, Ser-Glu, Glu-Glu, N-acetylglutamine, N-acetylglutamic acid, N5-ethylglutamine); and (iii) CoA/succinyl CoA divalents (Fig. 3D). Metabolomics profiling clearly demonstrated the accumulation of metabolites in a mutation-specific manner.

### Ribonucleoside di/triphosphate accumulation resulted from *rpoBC* mutations

**Fig 4:**
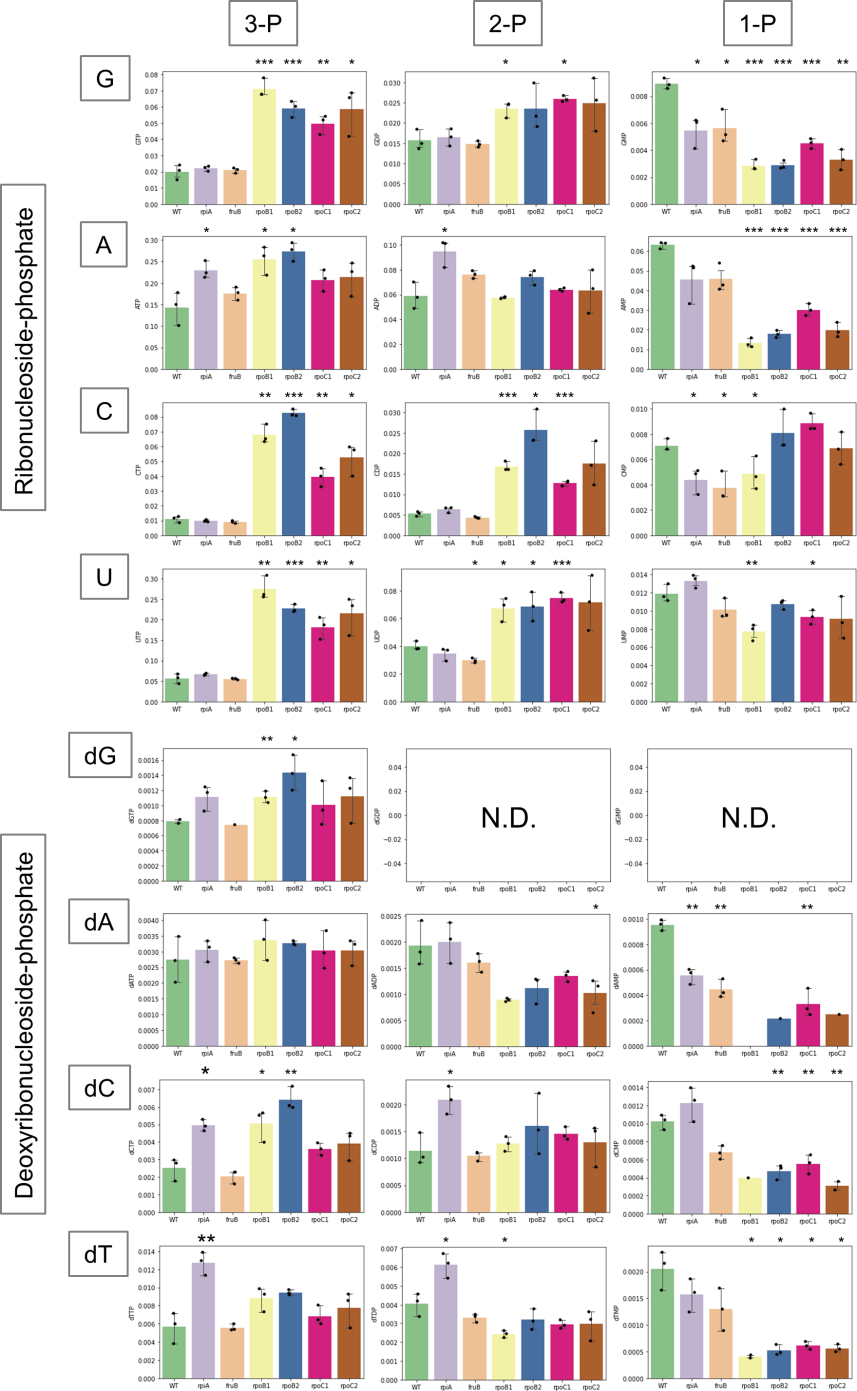
Ribo/deoxyribonucleotide profile of JMUB217-derived mutants. Quantitative values of each ribo/deoxyribonucleotide of JMUB217-derived mutants with reduced oxacillin susceptibility: *rpiA* (JMUB217-7 RpiA^A64E^), *fruB* (JMUB217-13 FruB^A211E^), *rpoB1* (JMUB217-23 RpoB^H929P^), *rpoB2* (JMUB217-22 RpoB^Q645H^), *rpoC1* (JMUB217-19 RpoC^G950R^), and *rpoC2* (JMUB217-24 RpoC^G498D^). The *y-*axis represents the amount of intracellular ribo/deoxyribonucleotide (pmol) in 1 mL of cell suspension at OD_600_ = 1. Data represent means with standard error from three independent experiments. N.D., not detected. Mean values of intracellular ribo/deoxyribonucleotide of mutant strains were compared with that of wild-type via one-way ANOVA. *P  <  0.05, **P  <  0.01, and ***P  <  0.001. Figures 3 to 5 and Fig. S1 were constructed from data of the same metabolomics analysis.

Network analysis of the metabolomics data revealed an accumulation of ribonucleoside di/triphosphate in the *rpoBC* mutants. The build-up of excess ribonucleoside di/triphosphate is consistent with the downregulation of purine/pyrimidine biosynthesis and nucleotide transporter genes observed through RNA-seq analysis of the *rpoBC* mutants (8). To obtain a comprehensive overview of nucleotide metabolisms in *rpoBC* mutants, the levels of intracellular ribonucleotide/deoxyribonucleotide in mutants with reduced oxacillin susceptibility are summarized in Fig. 4. Remarkably, GTP achieved 3.6-, 3.0-, 2.5-, and 2.9-fold greater accumulation in JMUB217-23 (RpoB^H929P^), JMUB217-22 (RpoB^Q645H^), JMUB217-19 (RpoC^G950R^), and JMUB217-24 (RpoC^G498D^), respectively (Fig. 4). CTP also exhibited a respective 6.2-, 7.5-, 3.6-, and 4.8-fold increase, while UTP showed 4.9-, 4.0-, 3.2-, and 3.8-fold increase in JMUB217-23, JMUB217-22, JMUB217-19, and JMUB217-24, respectively (Fig. 4). ATP was slightly accumulated in *rpoB* mutants but remained unaffected in *rpoC* mutants. On the contrary, intracellular ribonucleoside monophosphates, especially GMP and AMP, were reduced in the *rpoBC* mutants. In contrast to ribonucleotide, a modest intracellular accumulation of dGTP and dCTP was observed in the *rpoB* mutants, while most of the deoxyribonucleoside phosphates were either unchanged or decreased in the mutants with reduced oxacillin susceptibility. In short, the metabolomics results showed that *rpoBC* mutations stimulated intracellular ribonucleoside di/triphosphate accumulation in OS-MRSA.

### Global metabolic alterations resulted from *rpoBC* mutations

**Fig 5:**
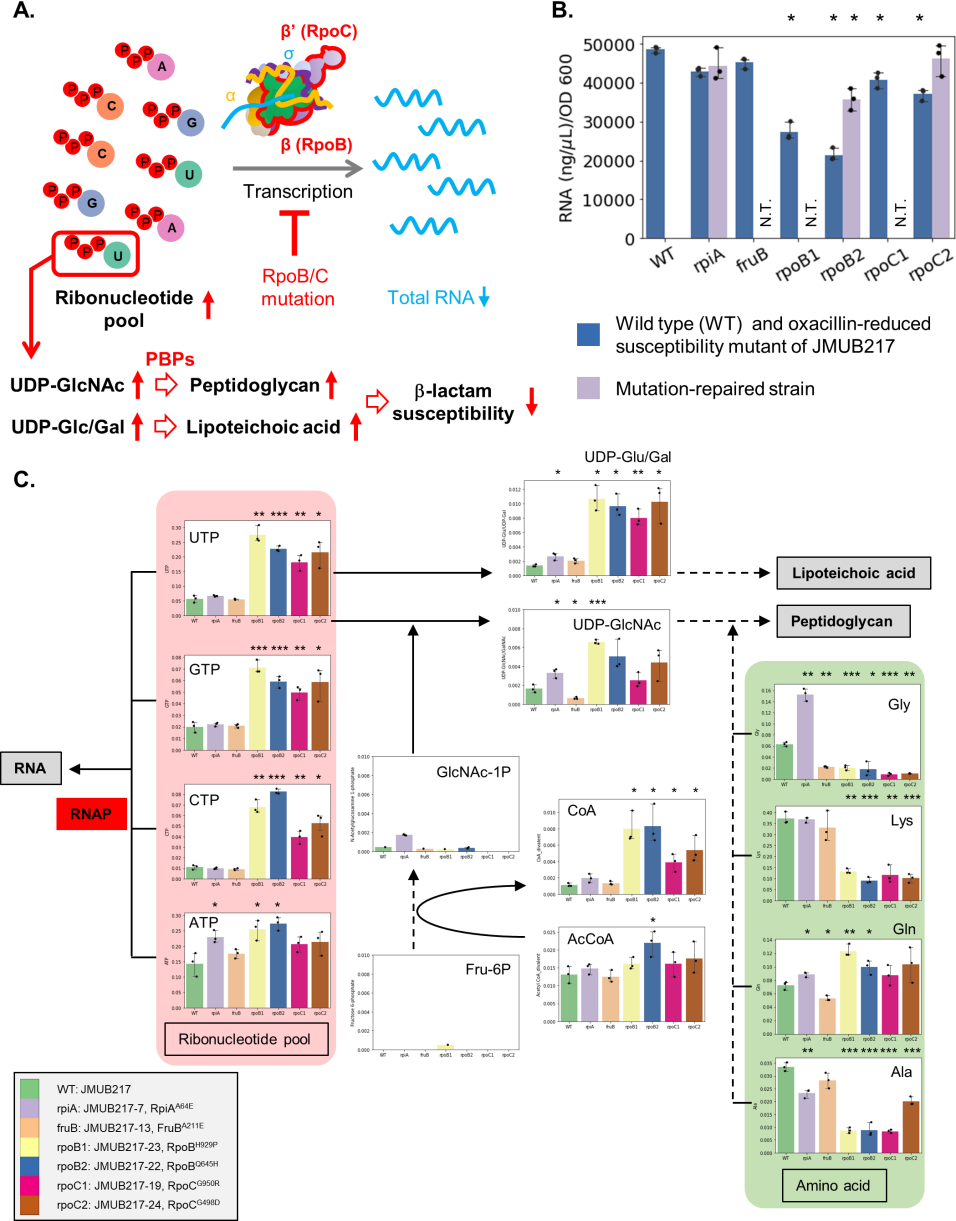
Metabolic alterations associated with *rpoBC* mutations. (**A**) Proposed mechanism of reduced β-lactam susceptibility mediated by *rpoBC* mutations. *rpoB/C* mutations cause dysregulation of RNAP transcription activity and a subsequent intracellular accumulation of ribonucleotides. Excessive accumulation of UTP enhances the production of UDP-GlcNAc and UDP-Glc/Gal, resulting in cell wall thickening and reduced β-lactam susceptibility in OS-MRSA. (**B**) The total amount of RNA in JMUB217-derived mutants grown to the mid-log phase (OD_600_ = 0.5). (**C**) RNA and peptidoglycan biosynthesis pathway of JMUB217-derived mutants. The *y*-axis represents the amount of intracellular metabolites (pmol) in 1 mL of cell suspension at OD_600_ = 1. Data represent means with standard error from three independent experiments. Mean values of intracellular metabolites of mutant strains were compared with that of wild-type via one-way ANOVA. *P  <  0.05, **P  <  0.01, and ***P  <  0.001. Figures 3 to 5 and Fig. S1 were constructed from data of the same metabolomics analysis.

The accumulation of ribonucleoside di/triphosphate is proposed to be a sequela of RNAP hypofunctioning caused by *rpoBC* mutations because ribonucleotides are building blocks of RNA (Fig. 5A). To elucidate the effect of *rpoBC* mutations on the transcription activity of RNAP, the total amount of RNA in mutants with reduced oxacillin susceptibility was measured. The mutations in *rpoBC* genes facilitated a substantial decrease in the levels of total RNA (Fig. 5B), whereby a respective 0.56-, 0.44-, 0.84-, and 0.76-fold decrease in total RNAs was observed in JMUB217-23, JMUB217-22, JMUB217-19, and JMUB217-24 (Fig. 5B). In addition, *rpiA* and *fruB* mutants also showed a slight decrease in total RNA. Thus, we deduced that *rpoBC* mutations reduced the general transcription activity of cells *in vivo*, thereby resulting in the accumulation of intracellular ribonucleoside di/triphosphate.

Nucleoside triphosphates (NTPs) are substrates of nucleoside diphosphate (NDP) sugars, which are important for the biosynthesis of many biological molecules, e.g., lipopolysaccharides, glycoproteins, and peptidoglycans. Therefore, excessive amounts of NTPs found in the *rpoBC* mutants are hypothesized to be consumed as NDP sugars and lead to peptidoglycan and lipoteichoic acid production (Fig. 5A). In this study, UDP-glucose/galactose (UDP-Glc/Gal; the two molecules could not be distinguished by CE-TOF-MS because they have the same molecular weight) and UDP-N-acetylglucosamine (UDP-GlcNAc) were measured by metabolomics analysis (Fig. 5C). As expected, intracellular UDP-Glc/Gal significantly increased in the *rpoBC* mutants, and UDP-GlcNAc (one of the important constituents of peptidoglycan) was significantly accumulated in JMUB217-23. In concordance with the accumulation of UDP-GlcNAc, three of the four amino acids, Gly, Lys, and Ala, required for peptidoglycan biosynthesis were significantly reduced in the *rpoBC* mutants, while their cellular levels of co-enzyme A divalent (CoA) were found to be increased.

**Fig 6:**
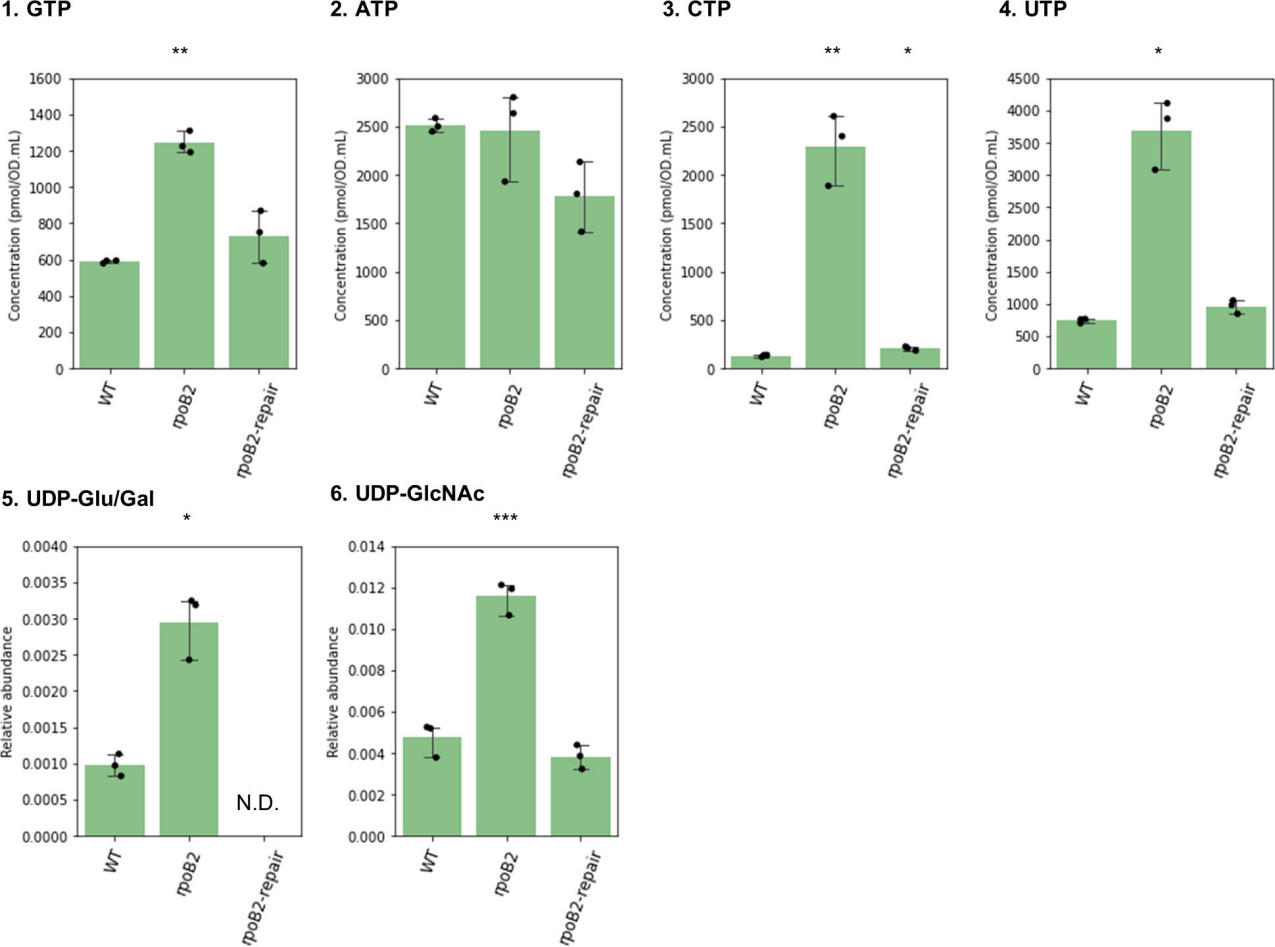
Intracellular levels of nucleoside triphosphates and UDP-carbohydrate of *rpoB* mutant and its mutation-repaired strain. Relative values of GTP, ATP, CTP, and UTP, and relative abundances of UDP-Glu/Gal and UDP-GlcNAc of JMUB217-derived mutant (*rpoB2*) and its mutation-repaired strain (*rpoB2*-repair). Mean values of intracellular metabolites of mutant/mutation-repaired strains were compared with that of wild type via one-way ANOVA. *P  <  0.05, **P  <  0.01, and ***P  <  0.001. Figure 6 was constructed using metabolomics data different from Fig. 3 to 5 and Fig. S1.

To confirm that the accumulation of NTPs and UDP-carbohydrates are mediated by RNAP gene mutations, we re-extracted metabolites of three representative studied strains: wild-type JMUB217, JMUB217-22 (*rpoB2*), and its mutation-repaired derivative, and their differential metabolomics profiles were analyzed using CE-TOF-MS. Our results showed that the accumulation of GTP/CTP/UTP, UDP-Glu/Gal and UDP-GlcNac in the JMUB217-22 was recovered in its mutation-repaired derivative to a level similar to that of the wild-type JMUB217 (Fig. 6). The consumption of peptidoglycan precursors in *rpoBC* mutants suggested that peptidoglycan biosynthesis in these cells could have been promoted.

### Ribonucleoside di/triphosphate accumulation reduced oxacillin susceptibility of *rpoBC* mutants

**Fig 7:**
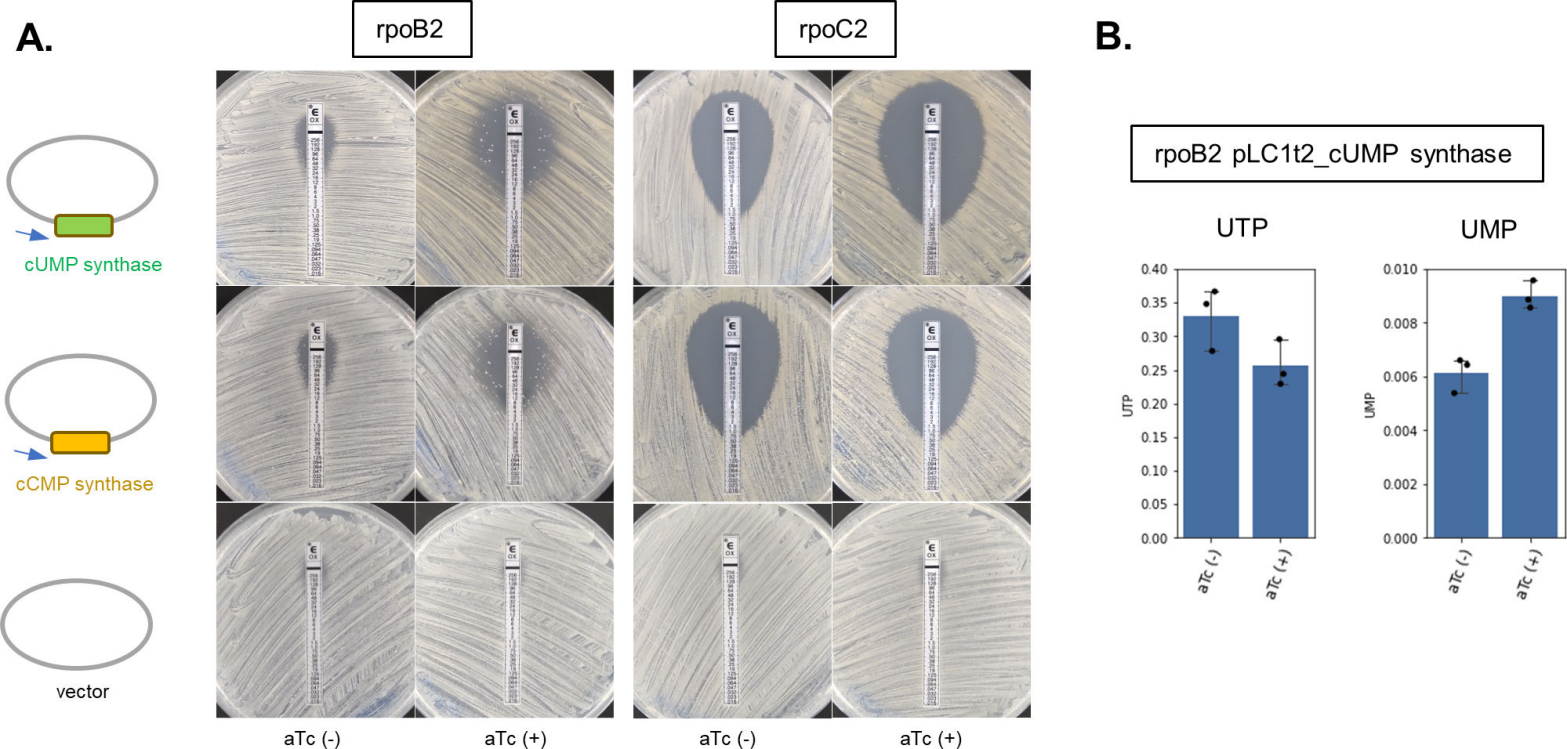
Altered oxacillin susceptibility by cUMP/cCMP gene expressions in JMUB217-derived *rpoBC* mutants. (**A**) Oxacillin susceptibility of cUMP/cCMP gene-expressing *rpoB2* and *rpoC2* mutants tested by E-test. Oxacillin susceptibility of *S. aureus* JMUB217-22 (*rpoB2*) and JMUB217-24 (*rpoC2*) carrying an aTc-inducible pLC1t2 plasmid containing JMUB217-derived cUMP and JMUB4998-derived cCMP synthase genes, respectively, was measured by oxacillin E-test. The concentration of aTc used for the induction was 50 ng/mL. (**B**) Relative abundances of intracellular UTP and UMP of *S. aureus* JMUB217-22 (*rpoB2*) harboring pLC1t2_cUMP synthase in the presence/absence of inducer, 50 ng/mL aTc. Data represent means with standard error from three independent experiments.

To confirm the causal relationship between oxacillin susceptibility and the accumulation of ribonucleoside di/triphosphate, *rpoBC* mutants expressing cyclic UMP (cUMP) and cyclic CMP (cCMP) synthase were constructed, and their oxacillin MIC was determined. cUMP and cCMP synthases encoded in Pycsar (pyrimidine cyclase system for antiphage resistance) were known to consume intracellular UTP and CTP for the generation of cUMP and cCMP, respectively (46), affecting the intracellular ribonucleotide di/triphosphate pool. JMUB217 was found to carry the cUMP synthase gene on the chromosome, though the effector gene was disrupted by the insertion of IS, while the cCMP synthase gene was detected in another OS-MRSA strain, JMUB4998 (8). In this study, JMUB217-22 (*rpoB*) and JMUB217-24 (*rpoC*) were, respectively, transformed with aTc-inducible pLC1t2 plasmid containing JMUB217-derived cUMP and JMUB4998-derived cCMP synthase genes. aTc induction increased oxacillin susceptibility in both transformants, suggesting that intracellular UTP and CTP can affect the oxacillin susceptibility of *rpoBC* mutants. The slight increase in oxacillin susceptibility without aTc induction is probably due to promoter leakage (Fig. 7).

### Metabolic alterations caused by *fruB* and *rpiA* mutations

FruB (1-phosphofructokinase) catalyzes the ATP-dependent phosphorylation of fructose-1-phosphate to fructose-1,6-bisphosphate (Fru-1,6P_2_) (47). Therefore, it is expected that a mutation in the *fruB* gene will affect the intracellular level of these two metabolites. Our results showed that fructose/glucose-1P (Fru/Glc-1P), which could not be distinguished by CE-TOF-MS due to having the same molecular weight, was accumulated in the *fruB* mutant, while intracellular Fru-1,6P_2_ was not affected by the *fruB* mutation (Fig. S1). We concluded that the FruB^A211E^ mutation is a loss-of-function mutation because of the intracellular accumulation of substrate Fru-1P. Most of the other metabolites were not altered by this mutation.

RpiA (ribose 5-phosphate isomerase A) is a key regulator of the pentose phosphate pathway, which catalyzes the conversion of D-ribose 5-phosphate to D-ribulose 5-phosphate (48). Similar to the case of the *fruB* mutation, the mutation of *rpiA* facilitated the accumulation of substrate ribulose-5P, while the product ribose-5P was not changed, indicating that the *rpiA* mutation induces loss of function (Fig. S1). However, in contrast to the *fruB* mutation, mutated *rpiA* broadly enhanced the accumulation of metabolites of the pentose phosphate and glycolysis pathways.

### Cell wall thickening in the mutants with reduced oxacillin susceptibility

**Fig 8:**
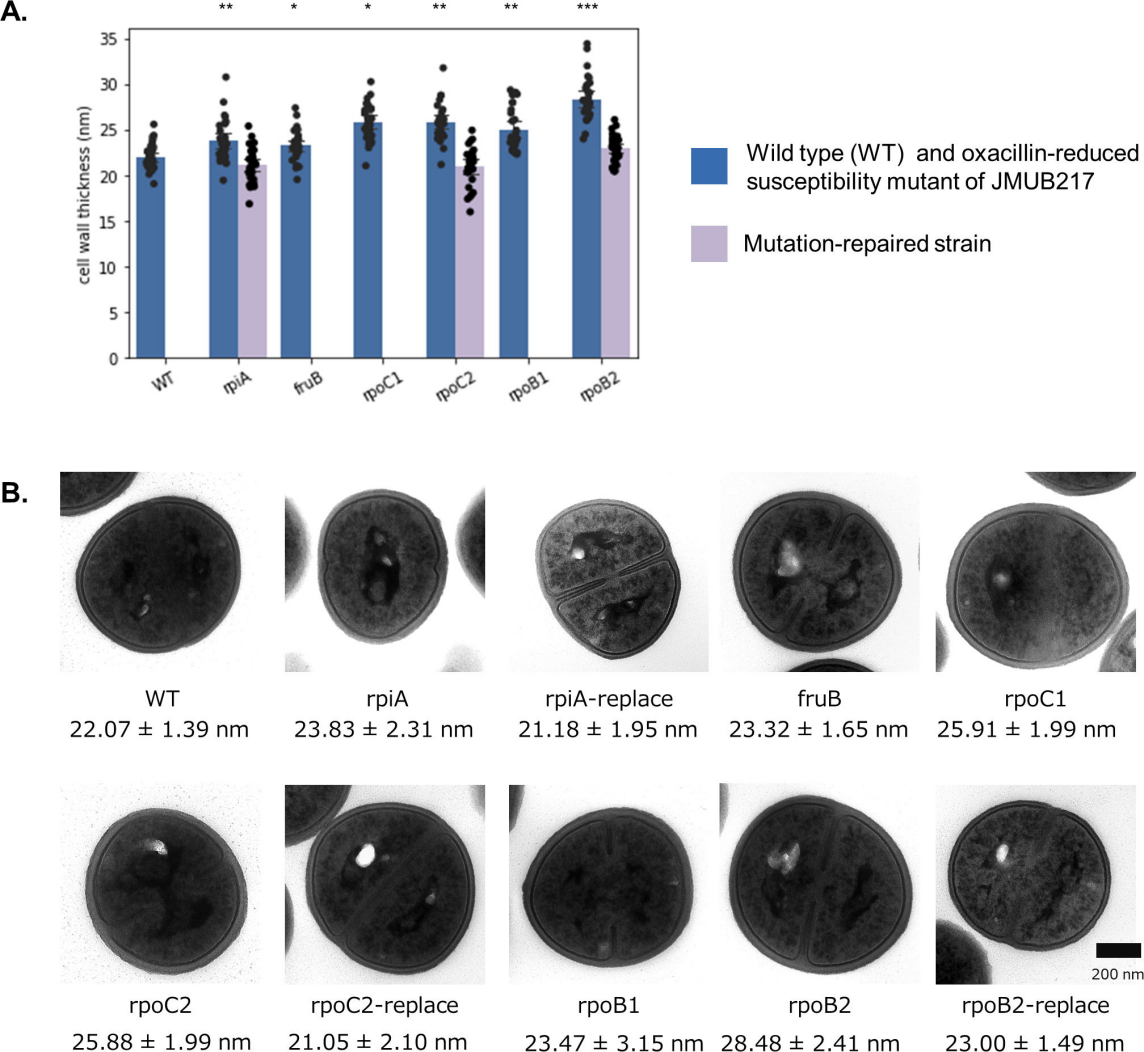
Cell wall thickness of JMUB217-derived mutants. (**A**) Cell wall thicknesses of 30 cells from each JMUB217-derived mutants with reduced oxacillin susceptibility and the mutation-repaired strains were measured by transmission electron microscopy. Mean values of cell wall thicknesses for JMUB217-derived mutants were compared with that of the wild-type via Student’s *t*-test. *P  <  0.05, **P  <  0.01, and ***P  <  0.001. (**B**) Transmission electron micrographs of representative JMUB217-derived mutants. Magnification, ×30,000.

The cell wall thickness of the mutants with reduced oxacillin susceptibility was determined in order to investigate the effect of *rpoBC* mutations on peptidoglycan biosynthesis, as our previous studies have shown alterations in the profile of intracellular metabolites associated with this process. Wild-type OS-MRSA JMUB217 had a cell wall thickness of 22.07 ± 1.39 nm, while the mutants carrying *rpoBC* mutations with reduced oxacillin susceptibility had significantly thicker cell walls (Fig. 8). The mean cell wall thicknesses were 25.91 ± 1.99, 25.88 ± 1.99, 23.47 ± 3.15, and 28.48 ± 2.41 nm in JMUB217-23 (RpoB^H929P^), JMUB217-22 (RpoB^Q645H^), JMUB217-19 (RpoC^G950R^), and JMUB217-24 (RpoC^G498D^), respectively. Interestingly, the cell walls of *rpiA* and *fruB* mutants were also shown to be thickened (23.83 ± 2.31 and 23.32 ± 1.65 nm, respectively).

## DISCUSSION

The emergence and spread of methicillin-resistant *Staphylococcus aureus* have become a major public health concern, as it can cause severe infections that are difficult to treat (1). OS-MRSA is a subtype of MRSA that is sensitive to oxacillin but still resistant to some β-lactam antibiotics (8–15). It has the potential to become highly resistant if exposed to β-lactam antibiotics, leading to treatment failure and further compounding the burden of diseases caused by *S. aureus*. Over decades, OS-MRSA have been isolated from various sources, including humans (both in the hospitals and community settings), animals, and food (8–15). Mutations in RNAP gene *rpoB* have been proposed as one of the key chromosomal mutations that contributed to the uniformly high β-lactam-resistant phenotype of OS-MRSA (49). Comparative genomics has revealed that the OS-MRSA JH1 strain acquired three to six mutations, including RpoB^D471Y, A473S, A477S, and E478D^, during the course of antibiotic treatment, and the resulting mutant derivative JH2 was rendered oxacillin-, vancomycin-, and rifampicin-resistant (40, 50). Another comparative genome analysis of 15 isolates retrieved from a persistent *S. aureus* infection also identified *rpoB* mutations as genetic determinants responsible for reduced staphylococcal susceptibility to rifampicin and oxacillin (41).

Mutations in *rpoB* are known to confer rifampicin resistance because β subunit of RNAP is the target of rifampicin (34). In addition to rifampicin, different *rpoB* and *rpoC* mutations have been reported to affect the susceptibility of *S. aureus* toward various other antibiotics, such as β-lactams, vancomycin, teicoplanin, linezolid, and daptomycin (21, 22, 25, 36–39, 51–53). *rpoB* mutations, N967I and R644H, caused phenotypic conversion of heterogenous-to-homogeneous and heterogeneous-to-Eagle-type β-lactam resistance, respectively (21). Another *rpoB* mutation, H481Y, was found to induce vancomycin resistance and promote heterogeneous vancomycin-intermediate *S. aureus* (hVISA)-to-VISA conversion (51, 54). Moreover, *rpoBC* mutations have been reported to be accompanied by other phenotypic changes such as prolonged doubling time, decrease in autolysis, and increased linezolid susceptibility, while associated with increased resistance to teicoplanin, vancomycin, and daptomycin (21, 51, 52, 54).

*rpoBC*-mutated strains included in this study showed reduced susceptibility to oxacillin and other β-lactam antibiotics, such as cefoxitin and imipenem. However, RNAP mutations did not render these strains resistant toward other cell wall synthesis inhibitors (vancomycin, teicoplanin, and fosfomycin) or rifampicin. Considering the distinct antibiotic susceptibility profiles displayed in these JMUB217-derived mutants, *rpoB* mutations carried in our studied strains are proposed to be associated with β-lactam resistance, while other gene mutations and/or different genomic backgrounds of MRSA might be the factor(s) regulating their glycopeptide susceptibility.

Although the association between mutated RNAP genes and reduced oxacillin susceptibility is well-demonstrated, the sequential events succeeding RNAP mutations that led to homogeneous high-level β-lactam resistance are not fully understood. Here, we unraveled the fundamental molecular mechanism linking RNAP gene mutation and staphylococcal β-lactam resistance through CE-TOF-MS-based metabolomics analysis.

Mutations in RNAP genes broadly affect the metabolic states of bacteria in adaptation to a variety of selection pressures, including antibiotics (8, 55–58), high temperature (59), starvation (60), and radiation (61). However, the *rpoBC* mutation-mediated metabolic alterations may differ between organisms. *Escherichia coli* frequently acquired mutations in the *rpoBC* genes in adaptation to glycerol minimum media (60). One of the mutants, which carries a 27-bp deletion in *rpoC,* demonstrated improved growth in glycerol media through reorganization of the metabolic network. This included an increase in redox cofactors NADH/NADPH and a decrease in most metabolites (tri/di-ribonucleotides) (62), which is in opposition to the case of OS-MRSA. In *E. coli*, the mutations in genes encoding RNAP are suggested to mimic the effect of a stringent response effector (p)ppGpp (60), which can directly bind to β′- and ω-subunits of RNAP, whereas in *Bacillus subtilis*, (p)ppGpp-mediated regulation is indirect and relies on cellular GDP/GTP ratio (63, 64), whereby depletion of cellular GTP is the trigger of the (p)ppGpp-mediated stringent response in Gram-positive bacteria (65). The reduction of oxacillin susceptibility in the *rpoBC* mutants might therefore not be directly related to (p)ppGpp-mediated response because our metabolome analysis shows an increase in intracellular GTP and undetectable (p)ppGpp level in the *rpoBC* mutants. On the other hand, rifampicin-treated *M. tuberculosis* showed dysregulation of nucleotide synthesis. Downregulation of purines (GTP, XTP, dATP, and dADP) and pyrimidines (CDP, dCDP, dCTP, and dTMP), as well as upregulation of ADP, cAMP, UTP, UDP, dUMP, and TDP nucleotides, were detected in *M. tuberculosis* following rifampicin exposure (66). The reported metabolomes were distinct from our *rpoBC*-mutated OS-MRSA strains, which showed an intracellular accumulation of ribonucleoside-di/triphosphate. *M. tuberculosis* with the *rpoB* mutation also showed a reduction in coenzyme A, while CoA was accumulated in our studied *rpoBC* mutants.

Nonetheless, certain *rpoB*-associated metabolic alterations persist across bacterial strains/genera. Exposure of *M. tuberculosis* to rifampicin is accompanied by degradation of mRNA (66). Likewise, *rpoBC* mutations were proposed in this study to reduce the general transcription activity of OS-MRSA as implicated by decreased total RNA levels. Apart from that, in concordance with the excessive amount of NTPs detected in our *rpoBC*-mutated OS-MRSA mutants, a laboratory-derived Mu3-6R strain carrying RpoB^R512P^ showed intracellular ribonucleotide accumulation in addition to slow growth phenotype and vancomycin resistance (39). Extracellular bases and nucleosides, such as inosine, uridine, guanine, cytidine, and guanosine, were also commonly found to be increased in *Streptomyces coelicolor* M1146 with *rpoB* mutation (67). Last but not least, increased peptidoglycan precursors and thickened cell walls were shown in the *rpoBC* mutants included in this study. It is interesting to note that these features were shared with *M. tuberculosis* carrying *rpoB* mutation in which alterations in metabolites associated with the maintenance of cell wall biosynthesis/cell wall remodeling and a paralleled change in cell wall structure were observable (68, 69).

Our previous study identified 141 mutations in 46 genes and 8 intergenic regions as potential genetic determinants of reduced oxacillin susceptibility (8). In this study, *rpiA* and *fruB* mutants (two other frequently mutated genes in OS-MRSA-derived mutants with reduced oxacillin susceptibility), in addition to *rpoBC* mutants, were analyzed. Our metabolomics analysis revealed that both *rpiA* and *fruB* mutations cause loss of function as implicated by the accumulation of substrates in the mutants. However, there is yet insubstantial experimental proof to support the deduction of the mechanism of reduced oxacillin susceptibility mediated through *rpiA* or *fruB* mutations. Further study is needed to warrant a more comprehensive understanding of the role(s) of *rpiA* and *fruB* in the acquisition of reduced oxacillin susceptibility in OS-MRSA.

This study revealed a novel mechanism for the acquisition of reduced β-lactam susceptibility in *S. aureus*. We proposed that mutations in *rpoB* and *rpoC* lead to the dysregulation of RNAP transcription activity and subsequent intracellular accumulation of ribonucleotides. This results in cell wall thickening and reduced β-lactam susceptibility in OS-MRSA. Mutations impacting nucleotide metabolisms [such as purine/pyrimidine metabolism, (p)ppGpp synthesis, and c-di-AMP signal] are often identified after cellular exposure to β-lactams, indicating their importance to β-lactam susceptibility. However, more studies are necessary to establish the intricate connection between chromosomal gene mutations and β-lactam susceptibility in *S. aureus*.

## MATERIALS AND METHODS

### Bacterial strains and growth conditions

The bacterial strains used in this study included one OS-MRSA parent strain JMUB217 (SCC*mec* type V, ST772, *blaI*-1 and -2 positive) and six of its representative derivative mutants with reduced susceptibility to oxacillin: JMUB217-7 (RpiA^A64E^), JMUB217-13 (FruB^A211E^), JMUB217-23 (RpoB^H929P^), JMUB217-22 (RpoB^Q645H^), JMUB217-19 (RpoC^G950R^), and JMUB217-24 (RpoC^G498D^) (8). All *S. aureus* strains were cultivated either in tryptic soy broth/agar (TSB/TSA; Becton Dickinson) or Mueller–Hinton broth/agar (MHB/MHA; Becton Dickinson) and incubated overnight at 37°C with constant agitation at 200 rpm if grown in broth. For plasmid propagation, *Escherichia coli* BL21 and DH5α strains were grown in Luria–Bertani (LB; Becton Dickinson) medium and incubated overnight at 37°C. To maintain plasmid pIMAY, 10 µg/mL chloramphenicol was added to the growth medium. All strains were stored at −80°C in 40% glycerol (Wako Pure Chemical, Japan) for preservation.

### Antibiotic susceptibility tests

The minimum inhibitory concentrations of oxacillin, cefoxitin, imipenem, vancomycin, teicoplanin, sulfamethoxazole/trimethoprim, fosfomycin, and rifampicin were determined using E-test method following the guidelines of the Clinical Laboratory Standard Institute (70).

### Construction of mutation-repaired strains

pIMAY-mediated allelic exchange (71) was conducted to construct mutation-repaired strains of JMUB217-7 (*rpiA*), JMUB217-22, (*rpoB*), and JMUB217-24 (*rpoC*) to confirm the effects of these mutations on oxacillin susceptibility. Briefly, native *rpiA*, *rpoB*, and *rpoC* of about 2 kbp each were amplified by pIMAY-N43rpiA-F/-R, pIMAY-N52rpoB-F/-R, and pIMAY-N75rpoC-F/R, respectively, using PRIME STAR MAX (TaKaRa, Japan) from template DNA of wild-type JMUB217. Using NEBuilder HiFi DNA Assembly (New England BioLabs, Inc.), each of the amplified fragment (native *rpiA*, *rpoB*, and *rpoC*) was cloned onto the pIMAY vector backbone linearized by PCR using a primer set of pIMAY-DW-F/pIMAY-DW-R2. The primers used for the construction of plasmids are listed in Fig. S1 and Table S2. The assembled plasmid DNAs were transformed into chemically competent *E. coli* DH5α, and the transformed cells were plated on LB agar with 10 µg/mL chloramphenicol. All three plasmids extracted from DH5α were then transformed into *E. coli* BL21 to improve their transformation efficiency into *S. aureus*. The plasmids extracted from BL21 were electroporated into the corresponding *S. aureus* mutants JMUB217-7, -22, or -24, and the cells were cultured at 30°C on TSA containing 10 µg/mL chloramphenicol. Single crossover was performed by growing overnight culture of transformants on TSA with 10 µg/mL chloramphenicol at 37°C. Then, double crossover was performed by incubating the single crossover mutants on TSA at 30°C. The double crossover event was confirmed by PCR and Sanger sequencing.

### Construction of cCMP and cUMP overexpression mutants

*S. aureus* JMUB217-22 (*rpoB*) and JMUB217-24 (*rpoC*) carrying aTc-inducible pLC1t2 plasmid cloned with JMUB217-derived cUMP and JMUB4998-derived cCMP synthase genes were constructed. At first, the SoxR terminator derived from pBTBX-2 was integrated into the pLC1 plasmid to decrease the downstream expression of an aTc-regulated inducible gene. SoxR terminator of 180 bp and a pLC1 backbone of 6 kbp were amplified by pBTBX2-F3/-R4 and pBTBX2-F3-pLC1dnF/pBTBX2-R4-pLC1upR, respectively, using PRIME STAR MAX and template DNA of pBTBX-2 (72) and pLC1 (73) plasmids. The PCR-amplified fragments were assembled by NEBuilder HiFi DNA Assembly generating pLC1t2. Next, cUMP and cCMP genes were, respectively, amplified by pLC1t2-N9_cUMPsyt-F1/R1 and pLC1t2-JMUB4998_cCMPsyt-F1/R1 from template DNA of wild-type OS-MRSA JMUB217 and JMUB4998 (8). The cUMP and cCMP gene fragments were then cloned with NEBuilder HiFi DNA Assembly onto the pLC1t2 vector backbone linearized by PCR using a primer set of pLC1t2-F-1/R-1. The assembled plasmid DNAs were transformed into competent *E. coli* DH5α cells, and then the extracted plasmids were subsequently transformed into *E. coli* BL21. The plasmids extracted from BL21 were electroporated into the corresponding *S. aureus* mutants JMUB217-22 or -24, and the resultant transformants were grown on TSA supplemented with 10 µg/mL chloramphenicol at 37°C. The expression of cUMP and cCMP genes were induced by a final concentration of 50 ng/mL aTC added to MHB/MHA media containing 2 µg/mL chloramphenicol.

### Metabolite extraction

Overnight cultures of parent *S. aureus* JMUB217 and mutant strains were diluted to OD_600_ = 1.0 with MHB. One milliliter of the culture was added to 50 mL of MHB, and the bacteria were incubated at 37°C with shaking until OD_600_ = 0.5. Then, the cultures were quickly chilled on ice for 15 min. Forty milliliters of the culture was transferred into a 50 mL tube, and the cells were collected by centrifugation at 5,800 × *g* for 5 min at 4°C. Pelleted cells were washed twice with Milli-Q water before being treated with 1,600 µL of methanol and ultrasonicated for 30 s to dissolve the pellet. The cell extract was subsequently treated with 1,100 µL of Milli-Q water containing internal standards [H3304-1002, Human Metabolome Technologies, Inc. (HMT), Tsuruoka, Yamagata, Japan] and left at rest for another 30 s. Cell extract containing spiked in internal standards was centrifuged at 2,300 × *g* for 5 min at 4°C, and 1,400 µL of supernatant was collected. The recovered supernatant was then centrifugally filtered through a Millipore 5 kDa cutoff filter (UltrafreeMC-PLHCC, HMT) at 9,100 × *g* for 120 min at 4°C to remove macromolecules, and the filtrate was finally centrifugally concentrated and resuspended in 50 µL of Milli-Q water for metabolome analysis at HMT. Three biological replicates were prepared for each OS-MRSA-derived mutant and parent strain.

### Metabolome analysis

Metabolome analysis was conducted by Basic Scan package of HMT using capillary electrophoresis time-of-flight mass spectrometry as described previously (45, 74). Briefly, CE-TOF-MS analysis was carried out using an Agilent CE capillary electrophoresis system equipped with an Agilent 6210 time-of-flight mass spectrometer (Agilent Technologies, Inc., Santa Clara, CA, USA). The systems were controlled by Agilent G2201AA ChemStation software version B.03.01 (Agilent Technologies, Inc.). Sample separations were carried out using fused silica capillary (50 µm i.d. × 80 cm total length) with commercial electrophoresis buffer (H3301-1001 and I3302-1023 for cation and anion analyses, respectively, HMT) as electrolyte. The spectrometer was scanned from *m*/*z* 50–1,000, and peaks were extracted using MasterHands, an automatic integration software (Keio University, Tsuruoka, Yamagata, Japan), to obtain peak information, which includes *m/z*, peak area, and migration time (MT) (75). Signal peaks corresponding to isotopomers, adduct ions, and other product ions of known metabolites were excluded. The remaining peaks were annotated according to the HMT metabolite database based on their *m/z* values with the MT determined by CE-TOF-MS. Areas of the annotated peaks were then normalized against internal standard levels and sample amounts in order to obtain the relative levels of each metabolite. Primary 110 metabolites were absolutely quantified based on one-point calibrations using their respective standard compounds. Hierarchical cluster analysis and principal component analysis (76) were performed by HMT’s proprietary MATLAB and R program, respectively. Detected metabolites were plotted on metabolic pathway maps using VANTED software (77). Network analysis was performed by Miru (Kajeka, UK) using Pearson correlation matrix with correlation coefficients of 0.85.

### Quantification of total RNA

Overnight cultures of the parent OS-MRSA JMUB217 and its derivative mutants with reduced oxacillin susceptibility/mutation-repair grown, respectively, in 1 mL of MHB (Becton Dickinson) were diluted to 1:100 in 10 mL of MHB and incubated at 37°C with constant agitation until OD_600_ of 0.5. The cells were harvested by centrifugation at 15,000 rpm for 1 min, resuspended in 600 µL of T_10_E_10_ buffer, pH 8.0 (10 mM Tris-HCl 8.0, 10 mM EDTA, pH 8.0), and lysed with a combination of 2 µL of 2 mg/mL lysostaphin (Sigma-Aldrich, USA) and 2 µL of achromopeptidase (FUJIFILM Wako Pure Chemicals, Japan) at 37°C for 5 min. To the lysed cells, 700 µL of acidic phenol saturated with 20 mM sodium acetate (NaOAc) and 60 µL of 3 M NaOAc (pH 4.8) were added. Consequently, total RNA was extracted using the phenol/chloroform method. The extracted RNA was treated with DNase I (Nippon Gene, CO., LTD.) and purified by acidic phenol/ethanol precipitation. The purified total RNA was finally dissolved in an appropriate volume of DEPC water, and the concentration was determined by NanoDrop (Thermo Fisher Scientific). Three biological replicates were prepared for each OS-MRSA-derived mutant, mutation-repaired strain, and parent strain.

### qRT-PCR

The extracted total RNA (500 ng per sample) was reverse transcribed into complementary DNA (cDNA) by PrimeScript 1st strand cDNA synthesis Kit (TaKaRa Bio, Japan). qRT-PCR was performed using TB Green Premix Ex Taq II (TaKaRa Bio, Japan). Primer sets used for the amplification of *mecA*, *purF,* and *guaA* are listed in Fig. S1 and Table S2. *rho* gene was used as the reference gene for normalization during gene expression analysis. The thermal cycling conditions included initial denaturation at 95°C for 30 s followed by 40 cycles of 95°C for 5 s and 60°C for 30 s. Three biological replicates were prepared for each strain.

### Transmission electron microscopy

To confirm the effects of metabolic alterations on peptidoglycan biosynthesis, cell wall thickness of parent OS-MRSA JMUB217 and its derivative mutants with reduced oxacillin susceptibility/mutation-repair was determined using the transmission electron microscope. Briefly, overnight cultures of the strains grown in MHB were passaged into fresh prewarmed MHB and incubated at 37°C with constant agitation until OD_600_ of 1.0. The cells were harvested by centrifugation at 10,000 rpm for 1 min at 4°C and resuspended with cold 0.1 M phosphate buffer, pH 7.4. Fixation, embedding, and staining of the samples were carried out following the methods described previously (37, 78, 79). The samples were visualized with transmission electron microscopy (Hitachi H-7600, Japan), and cell wall thicknesses were measured at nearly equatorial cut surfaces.

## SUPPLEMENTAL MATERIAL

Fig. S1 and Table S2 mbio.00339-24-s0001.docx Metabolic alteration with regard to central metabolomic pathways in JMUB217-derived mutants and primer list.

Table S1 mbio.00339-24-s0002.xlsx Metabolomics data.

ASM does not own the copyrights to Supplemental Material that may be linked to, or accessed through, an article. The authors have granted ASM a non-exclusive, world-wide license to publish the Supplemental Material files. Please contact the corresponding author directly for reuse.
