# Supplementary material for: Metabolic remodeling by RNA polymerase gene mutations is associated with reduced β-lactam susceptibility in oxacillin-susceptible MRSA
Source: mBio. 2024 May 2;15(6):e00339-24. doi: 10.1128/mbio.00339-24 (PMC11237739; doi:10.1128/mbio.00339-24)
Supplement: Fig. S1 and Table S2 — Metabolic alteration with regard to central metabolomic pathways in JMUB217-derived mutants and primer list. [file mbio.00339-24-s0001.docx]

**Supplement figure**

**Fig. S1. Metabolic alteration with regards to central metabolomic pathways in JMUB217-derived mutants**

The total amount of metabolites involved in glycolysis and pentose phosphate pathway of JMUB217-derived mutants. The *y*-axis represents the amount of intracellular metabolites (pmol) in 1 mL of cell suspension at OD_600_ = 1. Data represent means with standard error from three independent experiments. N.D., not detected. Mean values of intracellular metabolites of mutant strains were compared with that of wild-type (WT) via one-way ANOVA. *, *p* < 0.05, **, *p* < 0.01, ***, *p* < 0.001. Figs. 3-5 and S1 were constructed from data of the same metabolomics analysis.


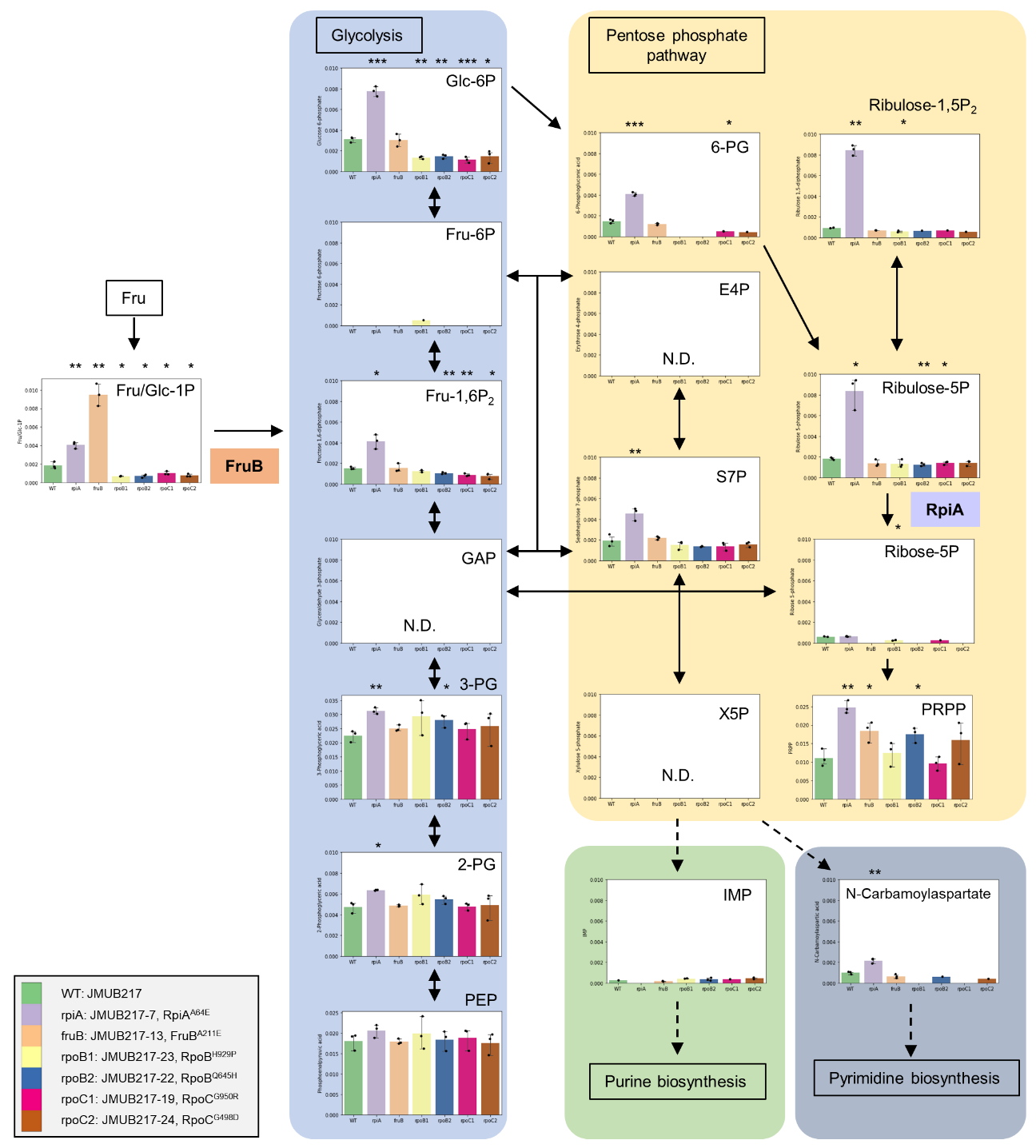


**Table S2. Primer list used in this study.**

| primer name | sequence | note |
| --- | --- | --- |
| pIMAY-DW-R2 | GCCGCTCTAGAACTAGTGGATC | construction for mutation replacement plasmid |
| pIMAY-DW-F | CGCCCTATAGTGAGTCGTATT | construction for mutation replacement plasmid |
| pIMAY-N52rpoB-F | GCTTGATCGTCGTAAAATCGAC | construction for mutation replacement plasmid |
| pIMAY-N52rpoB-R | CAGCCATACCTAAGTGTAGCTC | construction for mutation replacement plasmid |
| pIMAY-N75rpoC-F | TACCCAGGTCAATTCGTTGC | construction for mutation replacement plasmid |
| pIMAY-N75rpoC-R | TACCACAGTCTTCTTCACGAAC | construction for mutation replacement plasmid |
| pIMAY-N43rpiA-F | GAAACCTTGAAGAACTTGAGCC | construction for mutation replacement plasmid |
| pIMAY-N43rpiA-R | TATCGCGTCTTGTTGGTGAC | construction for mutation replacement plasmid |
| pIMAY-MCS-F | AAGAATAAACTGCCAAAGCAT | plasmid detection and confirmation of plasmid sequence |
| pIMAY-MCS-R | TGGTGTCCCTGTTGATACC | plasmid detection and confirmation of plasmid sequence |
| N52rpoB-F | AGGACCTGGTGGTTTAACACGTG | plasmid detection and confirmation of plasmid sequence |
| N52rpoB-R | CAATATGAATAGAAGTATACACGTC | plasmid detection and confirmation of plasmid sequence |
| N52rpoB-F2 | acgctatgacttagcaagcgtgg | confirmation for plasmid integration |
| N52rpoB-R2 | cacgagccataccagcttcttc | confirmation for plasmid integration |
| N75rpoC-F | GTAAACAAGGTCGTTTCCGTC | plasmid detection and confirmation of plasmid sequence |
| N75rpoC-R | AAGTCTTTCATACGGTCTAACATC | plasmid detection and confirmation of plasmid sequence |
| N75rpoC-F2 | gttgttgtagatccaggtccaac | confirmation for plasmid integration |
| N75rpoC-R2 | tcatcagtttcaggatgacgaattg | confirmation for plasmid integration |
| N43rpiA-F | AACACCCTCTTGAGTGCCTAC | plasmid detection and confirmation of plasmid sequence |
| N43rpiA-R | GTGAAACCAGTTGAAGATAACCATG | plasmid detection and confirmation of plasmid sequence |
| N43rpiA-F2 | tcgccatttcaacagcaatcg | confirmation for plasmid integration |
| N43rpiA-R2 | ggaatcaatagcatcagtggttatag | confirmation for plasmid integration |
| mecA-RTPCR-F | GAAGTAGAAATGACTGAACGTCCG | qRT-PCR |
| mecA-RTPCR-R | CGTTGCGATCAATGTTACCGTAG | qRT-PCR |
| purF-RTPCR-F | aagcagcgcaactaacatatatgg | qRT-PCR |
| purF-RTPCR-R | tcgttccatttgatcatctttaatcgc | qRT-PCR |
| guaA-RTPCR-F | tggtccaaattcagtttatgaagaagg | qRT-PCR |
| guaA-RTPCR-R | ccgtattcacgttcattggcac | qRT-PCR |
| rho-F-qRT-PCR-2 | tgaacgacttcagcagcttc | qRT-PCR |
| rho-R-qRT-PCR-2 | gaaatagcgaatgcaatcagtacg | qRT-PCR |
| pBTBX2-F3 | acgaactttaagaaggagatataccc | Construction for cCMP and cUMP overexpression plasmids |
| pBTBX2-R4 | cggtggccgcggaacaaaac | Construction for cCMP and cUMP overexpression plasmids |
| pBTBX2-F3-pLC1dnF | atctccttcttaaagttcgtAGCTTATTTTAATTATACTCTATCAATGATAGAGTGTC | Construction for cCMP and cUMP overexpression plasmids |
| pBTBX2-R4-pLC1upR | gttttgttccgcggccaccgGGGGATCCGTCGACCTGCAGCCAAG | Construction for cCMP and cUMP overexpression plasmids |
| pLC1t2-N9_cUMPsyt-F1 | tttaagaaggagatatacccATGAAAATAAGAGGCTATGATTACAAG | Construction for cCMP and cUMP overexpression plasmids |
| pLC1t2-N9_cUMPsyt-R1 | ttgaattcgttgacgaattctTTAATCAATCATACCTGACTTAATCCATTCATC | Construction for cCMP and cUMP overexpression plasmids |
| pLC1t2-JMUB4998_cCMPsyt-F1 | tttaagaaggagatatacccATGGACAATCACATAAAAATTTTTGACAATTTATTTC | Construction for cCMP and cUMP overexpression plasmids |
| pLC1t2-JMUB4998_cCMPsyt-R1 | ttgaattcgttgacgaattctTCAATCCTCTATTCCTAATTTTCTCACTTCTC | Construction for cCMP and cUMP overexpression plasmids |
